# Development and Validation of Near-Infrared Reflectance Spectroscopy Prediction Modeling for the Rapid Estimation of Biochemical Traits in Potato

**DOI:** 10.3390/foods13111655

**Published:** 2024-05-25

**Authors:** Paresh Chaukhande, Satish Kumar Luthra, R. N. Patel, Siddhant Ranjan Padhi, Pooja Mankar, Manisha Mangal, Jeetendra Kumar Ranjan, Amolkumar U. Solanke, Gyan Prakash Mishra, Dwijesh Chandra Mishra, Brajesh Singh, Rakesh Bhardwaj, Bhoopal Singh Tomar, Amritbir Singh Riar

**Affiliations:** 1 Division of Vegetable Science, The Graduate School, ICAR-Indian Agricultural Research Institute, New Delhi 110012, India; paresh.chaukhande@icar.gov.in (P.C.); manishamangal9@gmail.com (M.M.); jkranjan2001@yahoo.co.in (J.K.R.); 2 ICAR-Central Potato Research Institute Regional Station, Modipuram, Meerut 250110, India; skluthra@hotmail.com (S.K.L.); bchaukhande@gmail.com (P.M.); 3 Potato Research Station, SDAU, Deesa 385535, India; visitrnpatel@gmail.com; 4 ICAR-Indian Agricultural Research Institute, New Delhi 110012, India; siddhant.padhi1@gmail.com (S.R.P.); gyan.gene@gmail.com (G.P.M.); 5 ICAR-National Institute of Plant Biotechnology, New Delhi 110012, India; amol.solanke@icar.gov.in; 6 ICAR-Indian Agricultural Statistics Research Institute, New Delhi 110012, India; dwijesh.mishra@icar.gov.in; 7 ICAR-Central Potato Research Institute, Shimla 171001, India; birju16@gmail.com; 8 ICAR-National Bureau of Plant Genetic Resources, New Delhi 110012, India; 9 Department of International Cooperation, Research Institute of Organic Agriculture FiBL, 5070 Frick, Switzerland; amritbir.riar@fibl.org

**Keywords:** near-infrared reflectance spectroscopy (NIRS), nutritional variability, MPLS regression model, RSQ_external/internal_, RPD

## Abstract

Potato is a globally significant crop, crucial for food security and nutrition. Assessing vital nutritional traits is pivotal for enhancing nutritional value. However, traditional wet lab methods for the screening of large germplasms are time- and resource-intensive. To address this challenge, we used near-infrared reflectance spectroscopy (NIRS) for rapid trait estimation in diverse potato germplasms. It employs molecular absorption principles that use near-infrared sections of the electromagnetic spectrum for the precise and rapid determination of biochemical parameters and is non-destructive, enabling trait monitoring without sample compromise. We focused on modified partial least squares (MPLS)-based NIRS prediction models to assess eight key nutritional traits. Various mathematical treatments were executed by permutation and combinations for model calibration. The external validation prediction accuracy was based on the coefficient of determination (RSQ_external_), the ratio of performance to deviation (RPD), and the low standard error of performance (SEP). Higher RSQ_external_ values of 0.937, 0.892, and 0.759 were obtained for protein, dry matter, and total phenols, respectively. Higher RPD values were found for protein (3.982), followed by dry matter (3.041) and total phenolics (2.000), which indicates the excellent predictability of the models. A paired *t*-test confirmed that the differences between laboratory and predicted values are non-significant. This study presents the first multi-trait NIRS prediction model for Indian potato germplasm. The developed NIRS model effectively predicted the remaining genotypes in this study, demonstrating its broad applicability. This work highlights the rapid screening potential of NIRS for potato germplasm, a valuable tool for identifying trait variations and refining breeding strategies, to ensure sustainable potato production in the face of climate change.

## 1. Introduction

Potato (*Solanum tuberosum*) is an essential crop that is grown worldwide, with a production of 376.2 million tons spanning over an area of more than 16 million hectares [1]. Globally, India ranks second in potato production, with 54.23 million tons produced from an area of 2.24 million hectares [2]. Potatoes, along with cereals, provide food security to millions of people, owing to the high amount of carbohydrates, dietary fibers, vitamins, and minerals. The biochemical makeup of potatoes, which contain essential components such as starch (including amylose), protein, vitamin C, and antioxidants (phenols and carotenoids), is strongly linked to their nutritional value. These traits contribute to aesthetic appeal and offer numerous health benefits to humans [3].

The quality evaluation of potatoes is vital for crop development, nutritional research, and breeding programs. Traditional wet lab procedures have been used for a long time; however, these approaches have several drawbacks. These methods are often time-consuming, costly in terms of equipment and reagents, prone to human error, have a limited throughput, and are laborious and limited in obtaining real-time data, hindering rapid and efficient research analysis [4]. Additionally, conventional techniques usually entail destructive sampling, which makes it difficult to study the same sample again or to monitor changes over time [5].

Hence, there is an increasing need for effective, rapid, and non-destructive methods to evaluate the nutritional properties of largely consumed non-cereal crops, such as potatoes. NIRS is one such method and is a rapid and non-destructive analytical approach that has attracted significant interest in various food and feed industries. It is rapid in nature, making it possible to analyze a large number of samples in a short amount of time, with greater accuracy. In addition, NIRS model deployment requires very little sample preparation, eliminating the need for labor- and time-intensive processes. NIRS is a non-destructive technique that facilitates multiple measurements on a single material and streamlines the monitoring of changes. Moreover, it aligns with environmentally responsible practices by minimizing the need for chemical reagents and reducing sample waste, thereby highlighting its eco-friendly application [6].

NIRS involves examining matter interaction with electromagnetic radiation, typically using one or more wavelength bands within the range of 780–2500 nm. This radiation is directed at a sample, penetrating it and interacting with the molecular bonds, particularly -CH, -NH, -OH, and -CO formations, causing the absorption of light at their distinct vibration frequencies [7]. The spectrum produced, as a consequence, gives insightful information about the molecular makeup of the material. In spectroscopy evaluation, a crucial approach is spectrum calibration, which is achieved through multivariate regression methods. These techniques are collectively known as chemometrics, which are essential for calibrating near-infrared (NIR) spectra to wet chemistry values [8]. Due to the extensive range of organic compounds present in biomaterials, a precise calibration is essential. During the calibration process, the biochemical information present in a substance’s spectrum is separated from physical or chemical data, which are obtained through reference laboratory values [9]. The effectiveness of near-infrared spectroscopy (NIRS) relies on the complex connection between biochemical parameters and their corresponding absorption spectra. To establish accurate prediction models in NIRS, precise relationships must be established, which requires the pre-processing of spectral data using multivariate statistical analysis. Commonly used pre-treatment techniques include Multiplicative Scatter Correction (MSC) and Standard Normal Variate and Detrend (SNV-DT). To describe the relationship between biochemical components and spectral data, multivariate regression techniques such as partial least squares (PLS), modified PLS (MPLS), and Principal Component Regression (PCR) are employed [10].

The relevant body of work highlights the potential of NIRS for measuring the biochemical characteristics of several crops, including potatoes [11]. The first report of NIRS application in relation to potatoes was the evaluation of the moisture content in potato chips [12]. With advancements in NIRS techniques, researchers have investigated the use of NIRS in potatoes, to assess the starch and dry matter content [13,14], protein [15], phenol [9,16], carotenoids [3,17], fat [18], acrylamide [19,20], and moisture [21]. These studies provide insight into the adaptability of NIRS and demonstrate its potential to bring about a sea of change in potato quality evaluation, to strengthen Sustainable Development Goals 2 (Zero Hunger) and 3 (Good Health and well-being).

This investigation focused on the development and validation of NIRS techniques to provide an accurate and time-saving method for estimating the biochemical characteristics of potatoes. In addition, this study aimed to illustrate the potential benefits of NIRS in potato nutritional enrichment approaches and development by contrasting estimates obtained using NIRS with those obtained using more conventional wet lab approaches. Eight important nutritional traits, including vitamin C, total phenols, total carotenoids, anthocyanin, dry matter, starch, amylose, and protein, were assessed. These models can be used for the rapid screening of diverse potato germplasms, the use of nutritionally superior germplasms in potato breeding projects, and as an alternative to conventional wet lab methodology for the quality assessment of potatoes for sustainable production in the era of climate change.

## 2. Materials and Methods

### 2.1. Sample Collection

During *rabi* 2022–2023 potato growing season, 216 diverse tetraploid potato germplasms were planted in two different ago-climactic zones, namely, ICAR-CPRI RS, Modipuram, Meerut, Uttar Pradesh (29.06° N, 77.70° E) and Potato Research Station, SDAU, Banaskantha, Dessa, Gujarat (24.25° N, 72.18° E), following the augmented block design. Standard crop cultivation practices were adopted during the growing season. Mature tubers were harvested 90 days after planting. After harvesting, a marketable tuber (40–50 g, free from defects and damage) was preferred for nutritional evaluation.

### 2.2. Sample Preparation

The samples were thoroughly cleaned with tap water, without any contamination or loss of skin. Care was taken during sample selection for wet lab analysis, such as avoiding green color, external damage, and internal unwanted tuber flesh pigmentation. The composite samples were created by combining three to five tubers, with the number dependent on their size.

**Figure 1:**
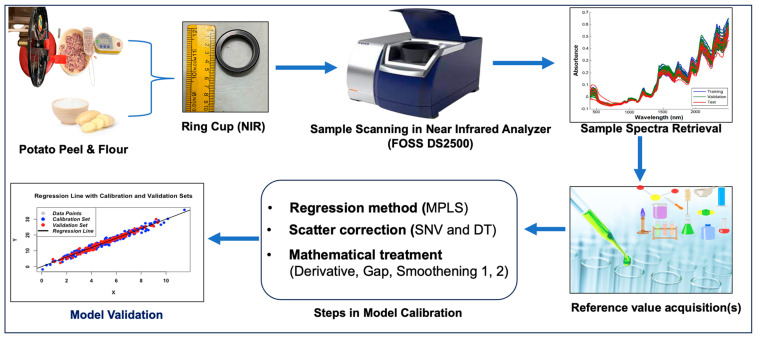
Schematic representation of NIRS model development and wet lab biochemistry of potatoes.

The tubers were peeled with a potato peeler, and the flesh of the selected tuber was quartered and placed in an NIR ring cup for scanning (Figure 1). The remaining flesh tissues of peeled tubers were used for the estimation of biochemical parameters, viz., vitamin C, total phenols, total carotenoids, anthocyanin, and dry matter. In addition, 50 g of tuber flesh from the same sample was kept in a hot air oven (103 ± 2 °C) for 12–24 h for uniform drying. The dried tuber tissue was then subjected to a further fine (1 mm sieve) powder form using a FOSS Cyclotec. Later, the powder form was subjected to wet lab analysis of the remaining biochemical parameters, that is, starch, amylose, and protein. In the present study, both potato flesh and flour were utilized for NIRS and wet chemistry analyses.

### 2.3. Selection of Samples for Assembling the NIRS Model

Using the FOSS NIRS DS-3 spectrophotometer, (FOSS, Nils Foss Allé 1, DK-3400 Hilleroed, Denmark) 432 germplasms from both locations were scanned and their reflectance spectra were recorded within the wavelength range of 400 to 2400 nm. Ward’s method of performing hierarchical clustering on normalized spectral data from 432 germplasms was used, with a squared Euclidean distance metric. The resulting clusters were further analyzed to identify the major clusters and sub-clusters. From this analysis, a subset of 120 highly diverse germplasms was subjected to wet lab analysis that represented the entire spectrum of variability within the dataset.

### 2.4. Generation of Reference Data for NIRS Prediction Models

#### 2.4.1. Vitamin C

Vitamin C estimation was performed using the Folin-phenol reagent method [22] on potato flesh. The absorbance at 630 nm was measured using a UV–VIS spectrophotometer and the results were expressed in milligrams per 100 g on fresh weight basis.

#### 2.4.2. Total Phenols

The Folin–Ciocalteu reagent assay, involving oxidation and reduction reactions, was used to assess the total phenolic content of potato flesh [23]. A UV–VIS spectrophotometer was used to measure the absorbance at 650 nm and the results are expressed in terms of gallic acid equivalents (GAEs) per 100 g of fresh weight.

#### 2.4.3. Total Carotenoids

The total carotenoid content in the flesh of potatoes was assessed using the AOAC method [24] and the absorbance at 420 nm was measured. The findings were expressed in µg per 100 g on a fresh weight basis.

#### 2.4.4. Anthocyanin

The total anthocyanin content in potato flesh was determined using the pH differential method using the AOAC Official Method 2005.02 [25]. The pH of the two solutions was set at 1.0 and 4.5 using concentrated hydrochloric acid, prior to taking the absorbance readings in multi-wavelength at 520 nm (HCl/KCl) and 700 nm (sodium acetate), respectively; the results were expressed in µg per g, as cyanidin-3-glucoside equivalents on fresh weight basis.2.4.5. Dry Matter

The dry matter content was determined using the AOAC method 925.10 [9]. Potato tuber tissue (50 g) was placed in an oven at 103 ± 2 °C until a constant weight was obtained. The dry matter percentage was calculated using the following formula: Dry matter (%) = [(final weight − crucible weight)/initial weight] × 100.

#### 2.4.5. Starch

The Megazyme total starch assay kit, which employs α-amylase, amyloglucosidase, and glucose oxidase peroxidase, was used to assess the total starch concentration in accordance with AOAC 996.11 [26]. A UV–VIS spectrophotometer was used to measure the absorbance at 510 nm and the results were represented as percentages based on a dry weight basis.

#### 2.4.6. Amylose

Amylose content from potato flour was estimated on a dry weight basis using a simplified method with a Continuous Flow Analyzer (CFA) in San++ Automated Wet Chemistry Analyzer Model 3000 (Skalar Analytical, Breda, The Netherlands) using the iodine method [27].

#### 2.4.7. Protein

The protein content of potato flour on a dry weight basis was determined using the Dumas combustion method [28]. The protein content was determined by converting the nitrogen percentage (%N) using the Jones conversion factor of 6.25.

### 2.5. Spectroscopic Analysis

A FOSS NIRS DS-3 spectrophotometer, which was equipped with Win ISI Project Manager Software Version 1.50, was used for spectroscopic analysis of both potato flesh and flour. Peeled potatoes were sliced using a rolling disc slicer, giving a 0.5 mm slice thickness. A disc of 3.7 cm in diameter was taken from the center of a slice to fit in the NIRS cuvette. The total moisture content was determined using AOAC 934.01 [29] and a range of 73–82.3% was observed in total of 120 potato germplasms. About 5 g of homogenized potato flour was filled in a cuvette and was scanned in a quartz window-equipped circular ring cup with a thickness of 1 mm and 3.8 cm. The check sample P/N:60053128, S/N:83924 provided by the instrument manufacturer, FOSS, was used for calibrating the instrument’s optical performance. An average spectrum was generated by subjecting a sample to 32 scans within the 400–2500 nm range, resulting in the recording of log (1/R) values at 2 nm increments. Here, “R” signifies reflectance.

#### 2.5.1. Development of Calibration and Validation Sets

A systematic approach was followed to create calibration and validation sets, involving a comprehensive assessment of 120 potato germplasms, consisting of local varieties, wild species, hybrids, CIP accessions, advanced breeding lines, and commercial varieties, all of which were evaluated for nutritionally relevant biochemical parameters using well-established biochemical protocols. Germplasms from both locations were considered in the calibration and validation sets for model development. The 120 germplasms were then carefully divided into a training set (calibration) comprising 80 samples and an external validation set comprising 40 samples. This categorization was based on the variations observed in the biochemical parameters of the samples. The values were meticulously organized using Microsoft Excel to ensure that both the training and validation sets had samples with similar variability and nearly equal minimum and maximum values. This strategic approach greatly facilitated the modeling process, with the validation set helping to guide predictions for the remaining germplasm.

#### 2.5.2. Calibration and Validation of Equations

The calibration equation was developed using Win ISI III Project Manager Software Version 1.50. Multivariate analysis was employed to regress the spectral data against laboratory values. Using software, MPLS regression, along with cross-validation, was carried out on the complete spectrum. Various mathematical approaches, including SNV–DT (SNV with detrend), have been used for scatter correction and spectral data pre-processing for each biochemical parameter [8]. A combination of mathematical treatments, denoted as “2,4,4,1”, “2,8,8,1”, “3,4,4,1”, and “3,4,3,2” were used in the model development process. In these notations, the first digit represents the order of the derivative, while the second digit signifies the gap. The third and fourth digits specify the number of data points employed in the first and second smoothing processes, respectively. The quality of the calibration equations was evaluated using various metrics, including the coefficient of determination (RSQ), the standard error of cross-validation [SEC(V)], the standard deviation (SD), and one-minus the variance ratio (1-VR) [6]. Additionally, the RSQ_external_, bias, SEP, SEP(C), and RPD values were used to gauge the model’s accuracy. The RPD values were particularly insightful, with their ranges indicating the reliability and quality of predictions.

## 3. Statistical Analysis

The Win ISI III Project Manager Software Version 1.50 was utilized as the platform for all calibration and prediction operations, which employed mathematical methods based on spectral and analytical data. The developed equations were used to monitor the reference and predicted values using the software. The accuracy and predictive capacity of the model were assessed using comprehensive statistical parameters, such as RSQ, slope, bias, RPD, and SEP(C). The coefficient of determination (internal/external) was depicted externally using the R programming software for statistical computing [30]. Using the Jamovi statistical software program v2.4, a paired sample *t*-test with a 95% confidence interval was carried out in order to ensure the significance of the findings [31].

## 4. Results and Discussion

### 4.1. Estimation of Nutritional Traits

**Figure 2:**
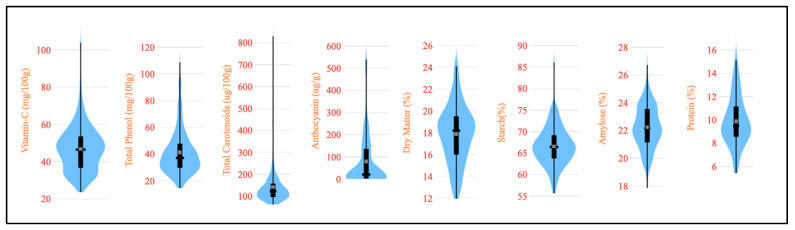
A violin plot depicting the nutritional variability among the studied 120 potato germplasm.

The evaluation of important nutritional traits for 120 diverse potato germplasms is summarized in Table 1, while the trait variability is illustrated in Figure 2 through violin plots. Our studied germplasm had a vitamin C content from 23.7 to 103.9 mg per 100 g on a fresh weight basis. This finding is in agreement with vitamin C evaluation in forty-eight Indian potato varieties from 19.4 to 58.4 mg per 100 g on a fresh weight basis [32]. The analyzed germplasms showed that the total phenol content varied between 14.5 and 108.7 mg per 100 g on a fresh weight basis. Previously, the total phenol content in four popular potato varieties was reported to range from 101.8 to 299.1 mg per 100 g [33]. We studied the total carotenoid content on a fresh weight basis among the germplasm and observed a range from 61.9 to 829.6 µg per 100 g on a fresh weight basis. Our reported range was wider than the results obtained by Tatarowska et al. for potato tubers from Poland [34]. However, the analysis of 152 potato germplasms in *S. phureja*, exhibited a broad range of total carotenoid contents, ranging from 103 to 2135 µg per 100 g on a fresh weight basis [3]. Our study evaluated germplasms for anthocyanin content and the trait ranged between 0.33 and 539 µg per g on a fresh weight basis. Similarly, an anthocyanin content of 11–174 mg per 100 g on a fresh weight basis was reported by Reyes et al. for purple- and red-fleshed potatoes from Texas, USA [35]. The dry matter content, an important biochemical attribute of potatoes, ranged from 11.95 to 24.11% among the studied germplasms. Similar findings, ranging from 14.1 to 35.2%, have been reported for potatoes [13,36]. Starch is also an important biochemical constituent and the results of our study, on a dry weight basis, showed variability from 55.7 to 86.1% among the germplasm. This range is consistent with the reported 83–90% in ten varieties and 69.4–72.3% in four varieties [37,38]. The range of protein content on a dry weight basis found in the germplasm was between 5.43 and 15.1%, confirming the reported range of 4.93 to 12.3% [36]. The amylose content varied from 17.9 to 26.7%, with several reports mentioning a range of 18–29% in potatoes on a dry weight basis [39,40].

### 4.2. NIRS Spectra Acquisition

**Figure 3:**
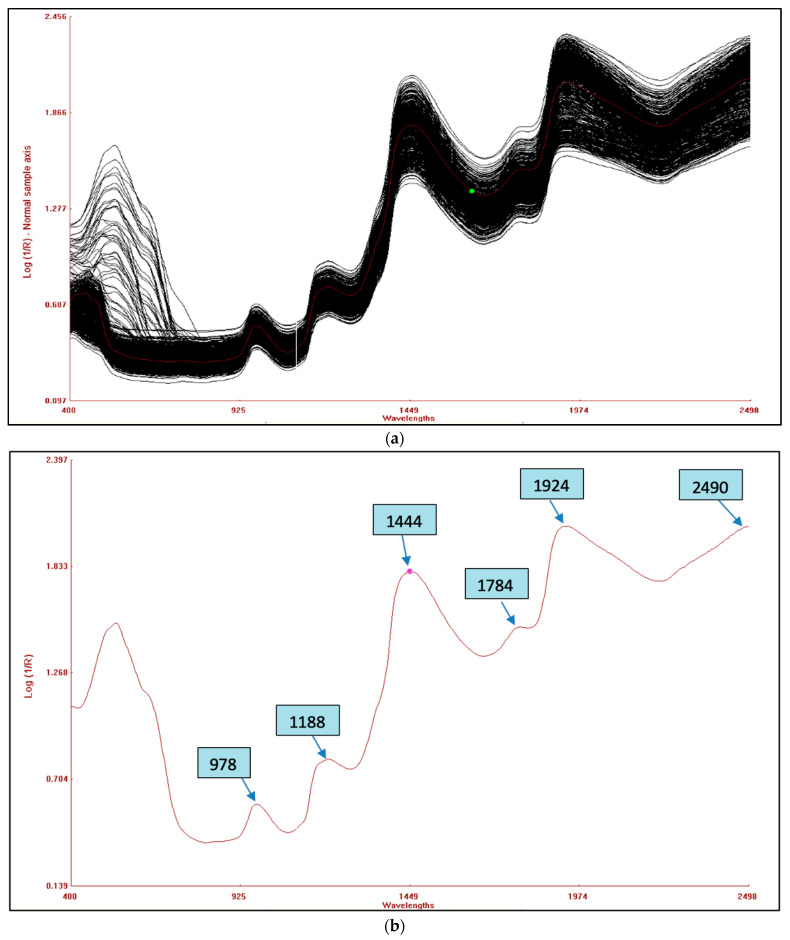

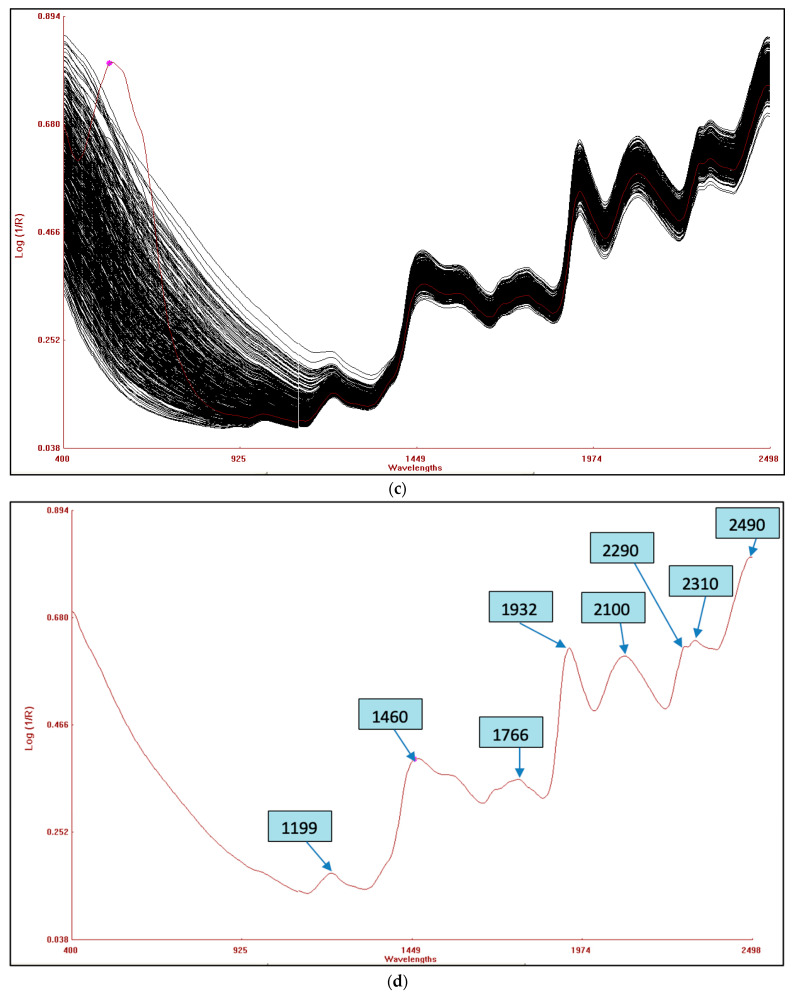
(**a**) Combined raw NIRS spectra of potato flesh of 120 germplasms. (**b**) An average reflectance spectrum of potato flesh with peaks. (**c**) Combined raw NIRS spectra of 120 potato flour germplasms. (**d**) Average reflectance spectrum of homogenized potato flour with peaks.

The combined raw NIRS spectra of the 120 germplasms based on potato flesh are shown in Figure 3a, whereas the raw spectra of potato flour are shown in Figure 3c. The bands that appear are as a result of the overlapping absorption that corresponds to the combination and overtones of vibrational modes N-H, O-H, and C-H found in proteins, fatty acids, and carbohydrates, respectively. Six primary absorption peaks in the fresh potato germplasm were detected at wavelengths of 978, 1188, 1444, 1784, 1924, and 2490 nm, as indicated in Figure 3b. However, for potato flour, eight absorption peaks were observed at wavelengths of 1199, 1460, 1766, 1932, 2100, 2290, 2310, and 2490 nm, as shown in Figure 3d. The spectral range between 2000 and 2222 nm revealed stretching vibrations related to C-O and N-H bonds, which are indicative of protein content [41]. In contrast, the bending and stretching of the O-H bonds in polysaccharides were detected around the peak at 1920 nm. This peak was due to the second overtone of O-H and C-O bending, associated with starch [42]. The 1650–1750 nm peak corresponds to the second overtone of O-H bending, which is primarily related to water. The first overtone of O-H stretching, related to hydroxyl phenol groups, was observed within the range of 1430–1470 nm. This range peak is as a result of the O-H functional group in starch, whereas the N-H peak is attributed to protein stretching in its first overtone. The 1180–1200 nm peak originates from the second overtone of the C-H vibrations associated with aliphatic hydrocarbons. Similar peaks were found in hot-dried and cold-dried samples of sweet potato [43] and in the spectra of potato chips [18].

### 4.3. Calibration of the NIRS Model

The process typically begins with the creation of a calibration set, also known as a training set, which serves to instruct and train the construction of the model. The use of regression algorithms, including MPLS, PLS, and PCR, can be implemented for the purpose of model development. In comparison with the PLS algorithm, MPLS is often considered to offer greater stability and accuracy [9], making it a good choice for this particular study. The MPLS technique utilizes both spectra and reference compositions to formulate equations, mitigating the influence of significant spectroscopic variations that may not be pertinent. Changes in absorption levels generally occur due to alterations in light scattering and path length, resulting from interventions with sample particles and light. Interpreting NIR spectra and creating a linear calibration becomes notably intricate because of these alterations [8]. Spectral pre-processing methods are applied to alleviate the compounding effects from particle size and scattering, specifically through scatter correction and derivatization techniques [21]. One such pre-processing technique, the Standard Normal Variate (SNV), involves mean removal from each spectrum, followed by normalizing each signal’s value by the standard deviation of the entire spectrum, to center it around zero. Additionally, a detrend (DT) approach was incorporated with the SNV, in this study, to rectify any shifts in the signal baseline and to reduce the NIRS signal noise. Table 2 presents a summary of the calibration developed for various potato biochemical parameters using the MPLS method for vitamin C, total phenols, total carotenoids, anthocyanin, dry matter from potato flesh, and starch, protein, and amylose derived from homogenized potato flour. To develop calibration equations for these parameters, various mathematical treatments, including “2,4,4,1”, “2,8,8,1”, “3,4,4,1”, and “3,4,3,2” were examined and finalized. The selection of the calibration equation was determined by the highest values of 1-VR and RSQ_internal_, as well as the lowest SEC(V) values. Improvements in the spectral resolution were accomplished using derivatives 2 and 3, which effectively eradicated baseline shifts and overlaid peaks. The use of gaps 4 and 8, as well as smoothing (S1, S2), helped to mitigate the impact of erratic high-frequency perturbations and to improve the signal-to-noise ratio within the specified spectral range. During the generation of the calibration equations, a small number of outliers (<10) resulting from scanning or analytical errors were identified and subsequently removed. RSQ_internal_ values for different biochemical traits, as shown in Table 2, were obtained for vitamin C (0.920), total phenols (0.893), total carotenoids (0.902), anthocyanin (0.837), dry matter (0.794), starch (0.644), amylose (0.905), and protein (0.986) for specific mathematical treatments, including “2,4,4,1”, “2,8,8,1”, “3,4,4,1”, “2,4,4,1”, “3,4,4,1”, “2,8,8,1”, “3,4,3,2”, and “3,4,4,1”, respectively.

### 4.4. Validation of the NIRS Model

External validation statistics for the evaluated biochemical traits are presented in Table 3. No outliers were removed in the external validation, to achieve a higher prediction power and ensure the robustness of the developed models. The best-fit models were chosen based on their higher RSQ_external_ and RPD values, as well as their low SEP, SD, slope, and bias values. To authenticate the model’s validity, the RPD value was used, which considers both SEP and variation in values and is more precise than SEP(C) [19]. Bedini et.al [10] found an RSQ_external_ of 0.90 for dry matter content, whereas our finding showed an RSQ_external_ of 0.89 (Table 3).

**Figure 4:**
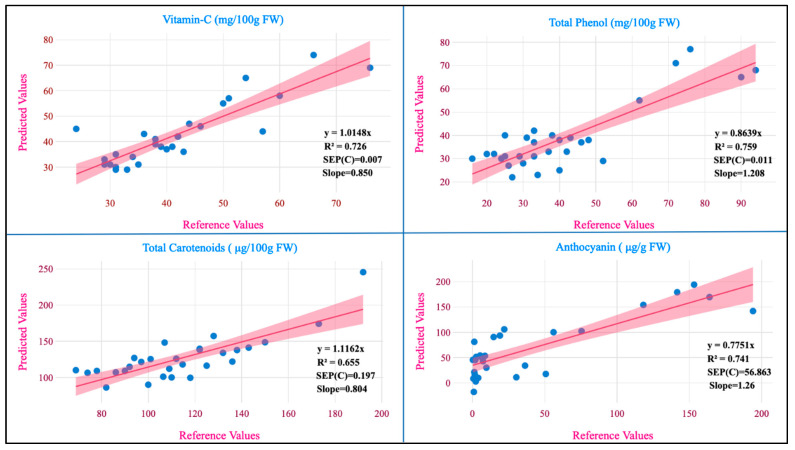

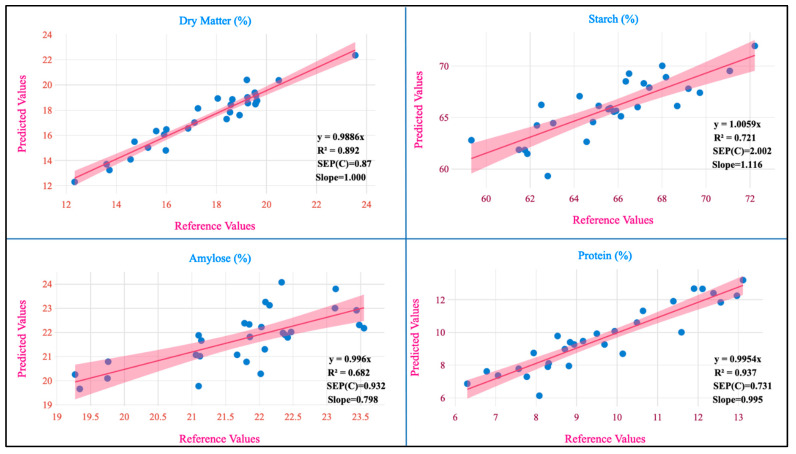
Measured references versus NIRS in the prediction set.

The regression plot of the predicted values against the reference values for the studied traits is shown in Figure 4.

The RPD values for the models developed from potato flesh for vitamin C, total phenol, total carotenoids, anthocyanin, and dry matter were 1.857, 2.00, 1.619, 1.856, and 3.041 and from potato flour for starch, amylose, and proteins were 1.867, 1.662, and 3.982, respectively. The RPD values serve as a measure of precision in MPLS models. When the RPD value is below 1.5, it signifies that the model lacks reliability. In the range of 1.5 to 2.0, it suggests the model’s capability to differentiate between high and low values. In the 2.0 to 2.5 range, it indicates an approximate ability for quantitative prediction. Falling between 2.5 and 3.0 signifies a good-quality prediction and if it exceeds 3.0, the prediction is considered excellent [44]. From this study, the RPD values for total phenol are in agreement with those reported (2.20) by Escuredo et al. [9] in potato. The RPD value for phenolics (2.00) indicates that the model is capable of differentiating between higher and lower values. The RPD value of dry matter (3.04) aligns with those reported (1.23–2.27) on various local and global models exclusively developed for dry matter [5]. The RPD value for the protein (3.98) is in close agreement with that reported (3.99) by Bernhard et al. [36]. In our study, RPD values were better in fresh samples than in flour samples; the same results were observed when assessing leaf nitrogen content in wheat [45]. These differences between the RPD values in our study may be due to the differences between the homogenization of fresh and flour samples. The slope represents the alteration in the estimated values, resulting from a unit change in the reference values. A slope value of 1 is ideal and any value close to 1 suggests an accurate model. The slope values for various traits in our study were vitamin C (0.85), total phenol (1.20), total carotenoids (0.80), anthocyanin (1.26), dry matter (0.89), starch (1.11), amylose (1.01), and protein (0.99). When assessing a model’s accuracy, one crucial factor to consider is its bias, which measures the degree of similarity between the predicted and reference values of the model [10]. The ideal bias value is zero, which is attained when the reference and predicted values are equal. This is considered the best possible outcome for bias. An underestimation model is signified by a negative bias and the overestimating model is signified by a positive bias [46]. The values of bias for different traits such as vitamin C (−0.001), total phenol (0.003), total carotenoids (−0.158), anthocyanin (−13.66), dry matter (−0.548), starch (−0.661), amylose (−0.053), and protein (0.004) where the developed models for six traits were found to be underestimating and two traits were found to be overestimating.

A paired *t*-test with a 95% confidence interval was used to assess if the mean of the dependent variable matched the analytical and predicted values for the investigated biochemical parameters [31]. In our findings, the *p-*value was greater than 0.05, indicating the precision and dependability of the models (Table 4). The *p-*values were as follows: vitamin C (0.429), total phenol (0.173), total carotenoids (0.115), anthocyanin (0.171), dry matter (0.190), starch (0.112), amylose (0.766), and protein (0.973). Therefore, the means of the NIRS method and the standard methods used to assess the traits were found to be not statistically different from one another.

## 5. Conclusions

This study aimed to create a rapid evaluation instrument for screening potato genetic resource collections and construct prediction models based on near-infrared reflectance spectroscopy (NIRS). MPLS-based regression models have been developed for vitamin C, total phenols, total carotenoids, anthocyanins, dry matter, starch, amylose, and protein, which are suitable for all traits. High RSQ_external_ and RPD values were found for most potato biochemical traits. The best models were developed for protein, followed by dry matter and total phenols, based on the RPD values. Compared with traditional wet lab techniques, these NIRS prediction models offer a more efficient, environmentally friendly, and less labor-intensive method to simultaneously evaluate the necessary components, providing desirable information about the biomolecules being studied. The combination of vibrational spectroscopy with multivariate methods in the models developed here has the potential to be a valuable analytical tool for potato breeding, food industry, and regulatory agencies. It can be used to efficiently and accurately develop, process, monitor, and evaluate the nutritional quality of potato in a cost-effective manner. This screening process aims to pinpoint trait-specific germplasm and select desired chemotypes, regardless of their genetic background, for the purpose of improving nutritional quality in potato breeding programs. However, these models can be forwarded to applicability studies to verify the accuracy and precision of the developed models.

## Figures and Tables

**Table 1:** Descriptive analysis of potato biochemical traits.

	Vitamin C (mg/100 g)	Total Phenol (mg/100 g)	Total Carotenoids (µg/100 g)	Anthocyanin (µg/g)	Dry Matter (%)	Starch (%)	Amylose (%)	Protein (%)
N	120	120	120	120	120	120	120	120
Mean	46.85	41.28	141.88	79.24	17.88	66.58	22.24	9.87
Std Dev	12.11	17.14	86.39	120.87	2.65	4.56	1.55	2.12
Maximum	103.89	108.69	829.60	539.04	24.11	86.09	26.74	15.12
Minimum	23.72	14.46	61.88	0.33	11.95	55.65	17.86	5.43

Note: Std Dev—standard deviation.

**Table 2:** Calibration statistics of biochemical traits evaluated in potatoes.

Traits	N	Outliers	Range	Math Treatment	Mean	RSQ	Slope	SD	SECV	1-VR
Vitamin C	80	3	10.40–81.00	2,4,4,1	45.70	0.920	1.030	0.011	0.008	0.524
Total Phenol	80	2	15.00–102.40	2,8,8,1	43.30	0.893	1.084	0.019	0.010	0.723
Total Carotenoids	80	2	8.27–239.52	3,4,4,1	123.90	0.902	1.001	0.385	0.291	0.422
Anthocyanin	80	7	33.40–342.98	2,4,4,1	69.18	0.837	0.993	0.912	0.517	0.683
Dry matter	80	4	9.16–26.57	3,4,4,1	17.86	0.794	0.946	2.904	1.793	0.620
Starch	80	8	56.79–75.61	2,8,8,1	66.28	0.644	0.987	2.841	1.992	0.505
Amylose	80	7	12.66–30.50	3,4,3,2	21.58	0.905	1.010	2.971	1.447	0.757
Protein	80	6	1.65–19.79	3,4,4,1	10.72	0.986	0.997	3.023	0.502	0.972

(Units: Vitamin C and Total Phenol: mg per 100 g, Total Carotenoids: µg per 100 g, and Anthocyanin: µg per g and Dry Matter (%) on fresh weight (FW) basis. Starch, amylose, and protein contents were expressed as percentages (%) on a dry weight basis. Abbreviations: N—number of samples, RSQ—coefficient of determination, SD—standard deviation, SECV—standard error of cross validation).

**Table 3:** Validation statistics of biochemical traits evaluated in potatoes.

Traits	N	Range	Math Treatment	Mean	RSQ	Slope	Bias	SD	SEP	RPD
Vitamin C	40	29.1–74.6	2,4,4,1	44.00	0.726	0.850	−0.001	0.013	0.007	1.857
Total Phenol	40	22.0–77.5	2,8,8,1	41.00	0.759	1.208	0.003	0.022	0.011	2.000
Total Carotenoids	40	86.1–245.6	3,4,4,1	113.50	0.655	0.804	−0.158	0.319	0.197	1.619
Anthocyanin	40	3.0–223.18	2,4,4,1	69.16	0.741	1.260	−13.66	105.51	56.86	1.856
Dry matter	40	12.30–24.02	3,4,4,1	17.88	0.892	1.000	−0.029	2.646	0.870	3.041
Starch	40	60.84–71.93	2,8,8,1	65.54	0.721	1.116	−0.661	3.737	2.002	1.867
Amylose	40	16.35–25.73	3,4,3,2	22.24	0.682	0.798	0.080	1.549	0.932	1.662
Protein	40	5.69–16.24	3,4,4,1	10.34	0.937	0.995	0.004	2.911	0.731	3.982

(Abbreviations: N—number of samples, RSQ—coefficient of determination, SD—standard deviation, SEP—standard error of performance, RPD—ratio of performance to deviation).

**Table 4:** Paired sample *t*-test at 95% confidence interval.

Pairs	Paired Differences					t Value	DF	*p* Value
	Mean	SD	SEM	95% Confidence Interval of the Difference				
				Lower	Upper			
Vitamin C reference—Vitamin C predicted	−1.18	−0.20	−0.03	−4.23	1.87	−0.806	40	0.429
Total Phenol reference—Total Phenol Predicted	3.04	6.10	1.19	−1.42	7.49	1.404	40	0.173
Total Carotenoids reference—Total Carotenoids predicted	15.8	0.20	0.04	−4.12	35.7	1.640	40	0.115
Anthocyanin reference—Anthocyanin predicted	−13.63	33.40	5.73	−33.47	6.21	−1.398	40	0.171
Dry matter reference—Dry matter predicted	0.02	0.86	−0.08	−0.15	0.74	0.402	40	0.687
Starch reference—Starch predicted	−0.66	0.90	0.17	−1.48	0.16	−1.649	40	0.112
Amylose reference—Amylose predicted	−0.07	0.26	0.04	−0.34	0.19	−0.585	40	0.562
Protein reference—Protein predicted	0.003	0.08	0.01	−0.21	0.22	0.034	40	0.973

## Data Availability

The original contributions presented in the study are included in the article, further inquiries can be directed to the corresponding authors.

## References

[1] FAO (2021). World Food and Agriculture-Statistical Yearbook-2021.

[2] Ministry of Agriculture & Farmers Welfare, Government of India (2021). Agriculture Statistics at A Glance.

[3] Bonierbale M., Grüneberg W., Amoros W., Burgos G., Salas E., Porras E., Felde T.Z. (2009). Total and individual carotenoid profiles in Solanum phureja cultivated potatoes: II. Development and application of near-infrared reflectance spectroscopy (NIRS) calibrations for germplasm characterization. J. Food Compos. Anal..

[4] Berger K., Machwitz M., Kycko M., Kefauver S.C., Van Wittenberghe S., Gerhards M., Verrelst J., Atzberger C., van der Tol C., Damm A. (2022). Multi-sensor spectral synergies for crop stress detection and monitoring in the optical domain: A review. Remote Sens. Environ..

[5] Wang S., Yan J., Tian S., Tian H., Xu H. (2023). Vis/NIR model development and robustness in prediction of potato dry matter content with influence of cultivar and season. Postharvest Biol. Technol..

[6] Gouveia C.S.S., Lebot V., Pinheiro de Carvalho M. (2020). NIRS Estimation of Drought Stress on Chemical Quality Constituents of Taro (*Colocasia esculenta* L.) and Sweet Potato (*Ipomoea batatas* L.) Flours. Appl. Sci..

[7] Ciurczak E.W., Igne B., Workman J., Burns D.A. (2021). Handbook of Near-Infrared Analysis.

[8] Maestresalas A.L. (2016). Near-Infrared Spectroscopy and Hyperspectral Imaging for Non-Destructive Quality Inspection of Potatoes.

[9] Escuredo O., Seijo-Rodríguez A., Inmaculada González-Martín M., Shantal Rodríguez-Flores M., Carmen Seijo M. (2018). Potential of near infrared spectroscopy for predicting the physicochemical properties on potato flesh. Microchem. J..

[10] Bedini G., Chakravartula S.S.N., Nardella M., Bandiera A., Massantini R., Moscetti R. (2023). Prediction of potato dry matter content by FT-NIR spectroscopy: Impact of tuber tissue on model performance. Future Foods.

[11] López A., Arazuri S., García I., Mangado J., Jarén C. (2013). A Review of the Application of Near-Infrared Spectroscopy for the Analysis of Potatoes. J. Agric. Food Chem..

[12] McDermott L.P. (1988). Near infrared reflectance analysis of processed foods. Cereal Foods World.

[13] Escuredo O., Meno L., Rodriguez-Flores M.S., Seijo M.C. (2021). Rapid Estimation of Potato Quality Parameters by a Portable Near-Infrared Spectroscopy Device. Sensors.

[14] Haase N.U. (2003). Estimation of dry matter and starch concentration in potatoes by determination of under-water weight and near infrared spectroscopy. Potato Res..

[15] López A., Arazuri S., Jarén C., Mangado J., Arnal P., Galarreta J.I.R.d., Riga P., López R. (2013). Crude Protein Content Determination of Potatoes by NIRS Technology. Procedia Technol..

[16] Shiroma-Kian C., Tay D., Manrique I., Giusti M.M., Rodriguez-Saona L.E. (2008). Improving the screening process for the selection of potato breeding lines with enhanced polyphenolics content. J. Agric. Food Chem..

[17] Valcarcel J., Reilly K., Gaffney M., O’Brien N. (2014). Total Carotenoids and l-Ascorbic Acid Content in 60 Varieties of Potato (*Solanum tuberosum* L.) Grown in Ireland. Potato Res..

[18] Palarea-Albaladejo J., Cayuela-Sánchez J.A., Moriana-Correro E. (2021). Estimating Fat Components of Potato Chips Using Visible and Near-Infrared Spectroscopy and a Compositional Calibration Model. Food Anal. Methods.

[19] Pedreschi F., Segtnan V.H., Knutsen S.H. (2010). On-line monitoring of fat, dry matter and acrylamide contents in potato chips using near infrared interactance and visual reflectance imaging. Food Chem..

[20] Segtnan V.H., Kita A., Mielnik M., Jorgensen K., Knutsen S.H. (2006). Screening of acrylamide contents in potato crisps using process variable settings and near-infrared spectroscopy. Mol. Nutr. Food Res..

[21] Ni Y., Mei M., Kokot S. (2011). Analysis of complex, processed substances with the use of NIR spectroscopy and chemometrics: Classification and prediction of properties—The potato crisps example. Chemom. Intell. Lab. Syst..

[22] Dani J.a. (1982). A New Calorimetric Technique for the Estimation of Vitamin C Using Folin Phenol Reagent. Anal. Biochem..

[23] Singleton V.L., Orthofer R., Lamuela-Raventós R.M. (1999). Analysis of total phenols and other oxidation substrates and antioxidants by means of folin-ciocalteu reagent. Methods Enzymol..

[24] Rodriguez-Amaya D.B. (2001). A Guide to Carotenoid Analysis in Foods.

[25] Lee J., Durst R.W., Wrolstad R.E. (2019). Determination of Total Monomeric Anthocyanin Pigment Content of Fruit Juices, Beverages, Natural Colorants, and Wines by the pH Differential Method: Collaborative Study. J. AOAC Int..

[26] Raatz S.K., Idso L., Johnson L.K., Jackson M.I., Combs G.F. (2016). Resistant starch analysis of commonly consumed potatoes: Content varies by cooking method and service temperature but not by variety. Food Chem..

[27] Molina L., Jimenez R., Sreenivasulu N., Cuevas R.P.O. (2019). Multi-Dimensional Cooking Quality Classification Using Routine Quality Evaluation Methods. Methods Mol. Biol..

[28] Waglay A., Karboune S., Alli I. (2014). Potato protein isolates: Recovery and characterization of their properties. Food Chem..

[29] Thiex N. (2009). Evaluation of Analytical Methods for the Determination of Moisture, Crude Protein, Crude Fat, and Crude Fiber in Distillers Dried Grains with Solubles. J. Aoac Int..

[30] RSoftware (2021). R: A Language and Environment for Statistical Computing.

[31] The Jamovi Project (2023). *jamovi* (Version 2.4) [Computer Software]. https://www.jamovi.org.

[32] Joshi A., Kaundal B., Raigond P., Singh B., Sethi S., Bhowmik A., Kumar R. (2021). Low-volume procedure to determine phytate and ascorbic acid in potatoes: Standardization and analysis of Indian cultivars. J. Food Compos. Anal..

[33] Makori S.I., Mu T.-H., Sun H.-N. (2021). Profiling of Polyphenols, Flavonoids and Anthocyanins in Potato Peel and Flesh from Four Potato Varieties. Potato Res..

[34] Tatarowska B., Milczarek D., Wszelaczyńska E., Pobereżny J., Keutgen N., Keutgen A.J., Flis B. (2019). Carotenoids Variability of Potato Tubers in Relation to Genotype, Growing Location and Year. Am. J. Potato Res..

[35] Reyes L.F., Miller J.C., Cisneros-Zevallos L. (2005). Antioxidant capacity, anthocyanins and total phenolics in purple-and red-fleshed potato (*Solanum tuberosum* L.) genotypes. Am. J. Potato Res..

[36] Bernhard T., Truberg B., Friedt W., Snowdon R., Wittkop B. (2016). Development of Near-Infrared Reflection Spectroscopy Calibrations for Crude Protein and Dry Matter Content in Fresh and Dried Potato Tuber Samples. Potato Res..

[37] Bach S., Yada R.Y., Bizimungu B., Fan M., Sullivan J.A. (2013). Genotype by environment interaction effects on starch content and digestibility in potato (*Solanum tuberosum* L.). J. Agric. Food Chem..

[38] Liu Q., Tarn R., Lynch D., Skjodt N. (2007). Physicochemical properties of dry matter and starch from potatoes grown in Canada. Food Chem..

[39] Ahmed S., Zhou X., Pang Y., Xu Y., Tong C., Bao J. (2018). Genetic diversity of potato genotypes estimated by starch physicochemical properties and microsatellite markers. Food Chem..

[40] Stawski D. (2008). New determination method of amylose content in potato starch. Food Chem..

[41] Plans M., Simó J., Casañas F., Sabaté J., Rodriguez-Saona L. (2013). Characterization of common beans (*Phaseolus vulgaris* L.) by infrared spectroscopy: Comparison of MIR, FT-NIR and dispersive NIR using portable and benchtop instruments. Food Res. Int..

[42] Padhi S.R., John R., Bartwal A., Tripathi K., Gupta K., Wankhede D.P., Mishra G.P., Kumar S., Rana J.C., Riar A. (2022). Development and optimization of NIRS prediction models for simultaneous multi-trait assessment in diverse cowpea germplasm. Front. Nutr..

[43] Su W.-H., Bakalis S., Sun D.-W. (2019). Chemometric determination of time series moisture in both potato and sweet potato tubers during hot air and microwave drying using near/mid-infrared (NIR/MIR) hyperspectral techniques. Dry. Technol..

[44] Chadalavada K., Anbazhagan K., Ndour A., Choudhary S., Palmer W., Flynn J.R., Mallayee S., Pothu S., Prasad K., Varijakshapanikar P. (2022). NIR Instruments and Prediction Methods for Rapid Access to Grain Protein Content in Multiple Cereals. Sensors.

[45] Ecarnot M., Compan F., Roumet P. (2013). Assessing leaf nitrogen content and leaf mass per unit area of wheat in the field throughout plant cycle with a portable spectrometer. Field Crops Res..

[46] Thyrel M., Aulin R., Lestander T.A. (2019). A method for differentiating between exogenous and naturally embedded ash in bio-based feedstock by combining ED-XRF and NIR spectroscopy. Biomass Bioenergy.

